# Clinical and Molecular Features of Epidermal Growth Factor Receptor (EGFR) Mutation Positive Non-Small-Cell Lung Cancer (NSCLC) Patients Treated with Tyrosine Kinase Inhibitors (TKIs): Predictive and Prognostic Role of Co-Mutations

**DOI:** 10.3390/cancers13102425

**Published:** 2021-05-17

**Authors:** Paolo Bironzo, Maria Lucia Reale, Tessa Sperone, Fabrizio Tabbò, Andrea Caglio, Angela Listì, Francesco Passiglia, Massimo Di Maio, Luisella Righi, Federico Bussolino, Giorgio V. Scagliotti, Silvia Novello

**Affiliations:** 1 Thoracic Oncology Unit, Department of Oncology, San Luigi Gonzaga Hospital, University of Torino, 10043 Orbassano, Italy; tessa.sperone@edu.unito.it (T.S.); fabrizio.tabbo@unito.it (F.T.); francesco.passiglia@unito.it (F.P.); giorgio.scagliotti@unito.it (G.V.S.); silvia.novello@unito.it (S.N.); 2 Medical Oncology Unit, Department of Oncology, Mauriziano Umberto I Hospital, University of Torino, 10128 Torino, Italy; andrea.caglio@edu.unito.it (A.C.); massimo.dimaio@unito.it (M.D.M.); 3 Pathology Unit, Department of Oncology, San Luigi Gonzaga Hospital, University of Torino, 10043 Orbassano, Italy; alisti@live.it (A.L.); luisella.righi@unito.it (L.R.); 4 Department of Oncology, University of Torino, 10060 Candiolo, Italy; federico.bussolino@unito.it; 5 Laboratory of Vascular Oncology, Candiolo Cancer Institute-IRCCS-FPO, 10060 Candiolo, Italy

**Keywords:** epidermal growth factor receptor (EGFR), non-small-cell lung cancer (NSCLC), tyrosine kinase inhibitors (TKIs), co-mutations, next-generation sequencing (NGS)

## Abstract

**Simple Summary:**

Co-mutations may affect EGFR-TKIs efficacy in advanced EGFR mutated NSCLC and could be associated with worse prognosis. Using a clinical next-generation sequencing (NGS) panel we retrospectively assessed the impact of co-alterations in 106 consecutive patients treated with front-line EGFR-TKIs. Clinical and molecular data were retrieved. According to our data, co-mutations do not seem to have any predictive nor prognostic role. Co-mutations are associated with younger age at diagnosis and lymph nodes metastases at baseline. No association with PD-L1 expression level was observed.

**Abstract:**

Background: Tyrosine kinase inhibitors (TKIs) show variable efficacy in epidermal growth factor receptor mutation-positive (EGFR+) NSCLC patients, even in patients harbouring the same mutation. Co-alterations may predict different outcomes to TKIs. Methods: We retrospectively analysed all consecutive EGFR+ advanced NSCLC treated with first-line TKIs at our Institutions. NGS with a 22 genes clinical panel was performed on diagnostic specimens. PD-L1 expression was also evaluated. Results: Of the 106 analysed specimens, 59 showed concomitant pathogenic mutations. No differences in OS (mOS 22.8 vs. 29.5 months; *p* = 0.088), PFS (mPFS 10.9 vs. 11.2 months; *p* = 0.415) and ORR (55.9% vs. 68.1%; *p* = 0.202) were observed comparing patients without and with co-alterations. Subgroup analysis by EGFR mutation type and TKIs generation (1st/2nd vs. 3rd) did not show any difference too. No correlations of PD-L1 expression levels by co-mutational status were found. Significant associations with presence of co-alterations and younger age (*p* = 0.018) and baseline lymph nodes metastases (*p* = 0.032) were observed. Patients without concomitant alterations had a significant higher risk of bone progression (26.5% vs. 3.3%, *p* = 0.011). Conclusions: Pathogenic co-alterations does not seem to predict survival nor efficacy of EGFR TKIs in previously untreated advanced NSCLC.

## 1. Introduction

Non-small-cell lung cancer (NSCLC) is the first cause of cancer-related death worldwide [1]. Epidermal growth factor receptor (EGFR) activating mutations identify a subgroup of patients deriving survival benefit from tyrosine kinase inhibitors (TKIs) [2]. Therefore, clinical guidelines suggest testing all advanced non-squamous NSCLC, as well as selected squamous cell lung cancer patients, for EGFR mutations along with other oncogenic alterations in order to provide a targeted treatment. However, clinical activity and efficacy of TKIs greatly vary between apparently similar patients, underlining a wide heterogeneity even in molecularly selected groups. Next-generation sequencing (NGS) technologies could evaluate multiple genes in parallel, enabling a deeper analysis of tumour genetics. Previous retrospective studies report conflicting results on the possible role of co-alterations in EGFR-mutation-positive (EGFR+) NSCLC (Table S1). However, these studies were extremely heterogenous in regard to ethnicity, type of treatment, sequencing technology. We retrospectively reviewed clinical and molecular data of EGFR+ patients treated at two Institutions to explore the role, if any, of pathogenic concomitant alterations as well as programmed death ligand-1 (PD-L1) expression.

## 2. Materials and Methods

### 2.1. Patient Demographics and Outcome Measurements

Patients with advanced NSCLC with activating EGFR mutations treated with TKIs at the Thoracic Oncology Unit of the AOU San Luigi Gonzaga (Orbassano) and, in case of first-line osimertinib, also at the Oncological Unit of the Mauriziano Umberto I Hospital (Turin) were included. EGFR mutations (exons 18–21) were detected by NGS (next generation-sequencing). Progression-free survival (PFS) was defined as the time interval from the start of EGFR TKIs treatment and disease progression or death. Overall survival (OS) was defined as the time from the start of cancer treatment and the patient’s death. Performance status (PS) was assessed according to the Eastern Cooperative Oncology Group (ECOG) score. The definition of treatment response and disease progression was determined by the investigators using the response evaluation criteria in solid tumours (RECIST) version 1.1. The date of the last follow-up corresponded to 30 January 2021. Demographic data, data on smoking history, PS and clinical outcomes were collected from medical records. The database used for the study included the following variables:

- Patient characteristics: age, sex, smoking habit
- Disease characteristics: date of first diagnosis, method and site of diagnosis, histology, staging (TNM VIII edition), date of diagnosis of metastatic disease, number and sites of metastases;
- Molecular characteristics: EGFR mutation type (exon 19 deletion, exon 21 L858R mutation, non-T790M exon 20 mutations, exon 20 T790M mutation, others), diagnostic technique, PD-L1 expression level, presence/absence of co-mutations;
- Clinical course: starting date of first-line treatment, PS, best response, suspension of therapy and motivation, progression of the disease with relative sites, local treatments (radiotherapy, surgery or other), site and result of biopsy at the time of progression (liquid and /or tissue). Similar data were collected for second and third lines of therapy;
- Survival outcome: date of the last follow-up, status of patients (dead or alive).

Patients treated with drugs other than single-agent first-line EGFR TKI, such as combination therapies in clinical trials, as well as patients with resistant mutations such as EGFR exon 20 insertions were excluded.

### 2.2. NGS Sequencing

NGS sequencing was performed using the Ion Torrent platform (ThermoFisher Scientific) with Oncomine solid tumour DNA and RNA kit assays allowing both the analysis of coding sequence variants of 22 genes (including *EGFR, ALK, ERBB2, ERBB4, FGFR1, FGFR2, FGFR3, MET, DDR2, KRAS, PIK3CA, BRAF, AKT1, PTEN, NRAS, NTRK, MAP2K1, STK11, NOTCH1, CTNNB1, SMAD4, FBXW7, TP53*) and the identification of 4 gene rearrangements (i.e., *ALK, RET, ROS1, NTRK1*). After sample adequacy assessment, tumour DNA or RNA was extracted by automated purification kits. The amplicon libraries were prepared with Ion AmpliSeq Library Kit (Thermo Fisher, Waltham, MA, USA). After PCR amplification and barcodes ligation, the amplicon libraries were equalized and pooled in an equal molar ratio. Emulsion PCR and template preparation were performed using Ion OneTouch Template Kit and Ion OneTouch system (Thermo Fisher) and sequenced. Data analysis was conducted automatically by Ion Reporter Software. Post-sequencing bioinformatics analysis matched the complementary strands of each barcoded DNA fragment to remove false-positive results. The variant allele fraction (VAF) was computed as the number of mutated DNA molecules divided by the total number (mutated plus wild type) of DNA fragments at that allele; variants have been called if the variant frequency was ≥5%. Only pathogenic or likely pathogenic variants were included in analyses based on the current literature.

### 2.3. Immunohistochemical Scoring for PD-L1

Immunohistochemical (IHC) detection of tumour PD-L1 expression was performed using the PD-L1 Clone 22C3 kit (pharmDx, Agilent Technologies, Santa Clara, CA, USA) and the Dako Omnis platform (Agilent Technologies, Carpinteria, CA, USA). The percentage of tumour cells with PD-L1 expression (positive membrane staining) was obtained by counting at least 100 viable cells, and this was the so-called TPS. The evaluation of PD-L1 expression followed the specific requests of the treating clinician in terms of selection of the tested cohort and timing (diagnostic biopsy or rebiopsy).

### 2.4. Definitions

Based on recent literature, patients were defined as having co-mutations if they harboured mutations of pathogenic or unknown significance according to the COSMIC database and the FATHMM-MKL algorithm (available at https://cancer.sanger.ac.uk/cosmic, accessed on 15 April 2021). Therefore, patients with co-occurring benign mutations were considered without co-mutations. An exploratory analysis considering all patients with co-alterations (pathogenic or not) was also performed.

### 2.5. Statistical Analysis

Descriptive analyses such as medians, intervals, frequencies, percentages were used to describe the baseline characteristics of the patients. The χ^2^ test was used to analyse the differences between clinical and genetic parameters of patients. The survival curves were estimated using the Kaplan–Meyer method and the log-rank test was used to determine the differences in the survival curves between the groups. A *p* <0.05, with 2-sided testing, was defined as statistically significant. Cox proportional hazard regression models were used to evaluate the association between co-mutations (present/absent) and PFS and OS, obtaining hazard ratios and 95% confidence intervals (CI). The analyses were carried out with SPSS Software (IBM corporation, Armonk, NY, USA).

## 3. Results

### 3.1. Patients’ and Tumour Characteristics

A total of 147 patients with advanced EGFR positive NSCLC treated with first-line EGFR TKIs between January 2015 and January 2020 were identified. A total of 14 patients were not eligible due to lack of diagnostic tissue for analysis, presence of exon 20 resistant mutations, or treatment other than single-agent first-line EGFR TKIs. Therefore, 133 patients treated with first-line single-agent EGFR TKI were included, 106 of them with complete molecular information (Figure S1). Clinical characteristics are reported in Table 1.

**Figure 1:**
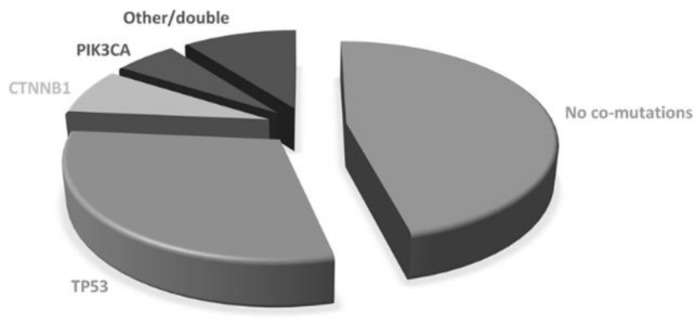
Baseline co-mutations.

The majority of patients were women (69/106; 65.1%) and never smokers (61/106; 57.5%). The median age was 69.8 years (range 32–90.7). All but one patient had adenocarcinoma histology. Most patients had stage IV disease at diagnosis (94/106, 88.7%) with one (35, 33.0%), two (29, 27.4%) or more (41, 38.7%) metastatic sites. Lymph nodes, bone and pleura were the most frequent metastatic sites at diagnosis (50%, 48.1% and 44.3%, respectively). ECOG PS at diagnosis was 0 in 48 patients (45.3%) and 1 in 50 (47.2%). Most patients were diagnosed with an EGFR exon 19 deletion (66, 62.3%) or L858R exon 21-point mutation (33, 31.1%), while others had uncommon or double mutations (see Supplementary Material). A total of 65 patients (61.3%) were treated with upfront first generation (gefitinib or erlotinib) or second-generation (afatinib) TKIs, while 41 (38.7%) received first-line osimertinib. Using diagnostic specimen, 57 patients (53.8%) showed co-occurring genetic alterations. The most common co-mutated genes were: *TP53* (*n*: 36, 34.0%), *CTNNB1* (*n*: 8, 7.5%), *PIK3CA* (n:6, 5.7%), while the others included *NRAS*, *MET*, *PTEN*, *AKT*, *SMAD4*, *RET*, *DDR2*, *FGFR3* (Figure 1). Double co-mutations occurred in 4 cases. According to the COSMIC database, 28 pathogenic TP53 mutations were found, while the others were benign or of neutral/unknown significance. Therefore, as for survival analysis, patients with concomitant mutations were defined by the presence of pathogenic mutations only. Using such a definition, 47 patients were considered co-mutation positive and 59 co-mutation negatives.

A greater proportion of patients with EGFR exon 19 deletion harboured concomitant mutations (*n*:33; 70.2%) as compared with those with EGFR exon 21 L858R mutation (*n*:10; 21.3%), although the difference was not statistically significant (*p* = 0.138) (Table 1, Table S2). No associations were found between the presence of concomitant mutations and gender or smoking habits. However, patients with concomitant mutations were younger than those without concomitant mutations (*p* = 0.018) (Table 1). The presence of concomitant mutations was associated with the presence of lymph node metastases at baseline (*p* = 0.032), while patients without concomitant mutations were more likely to present with pleural metastases (*p* = 0.007) (Table 1).

### 3.2. Treatment Outcomes in the Considered Cohort

**Figure 2:**
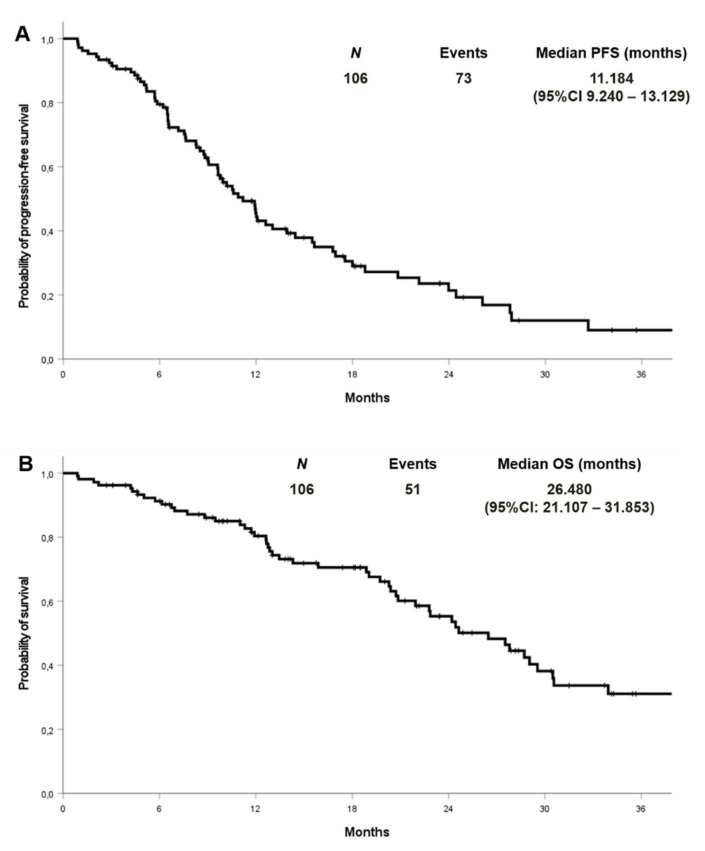
Progression-free survival (**A**) and overall survival (**B**) in patients with known co-mutational status.

**Figure 3:**
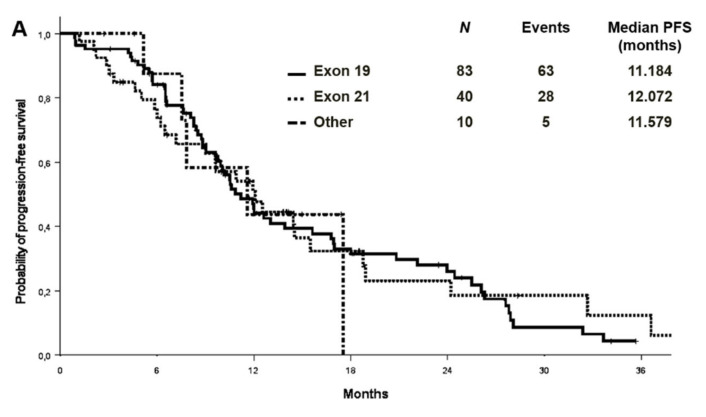

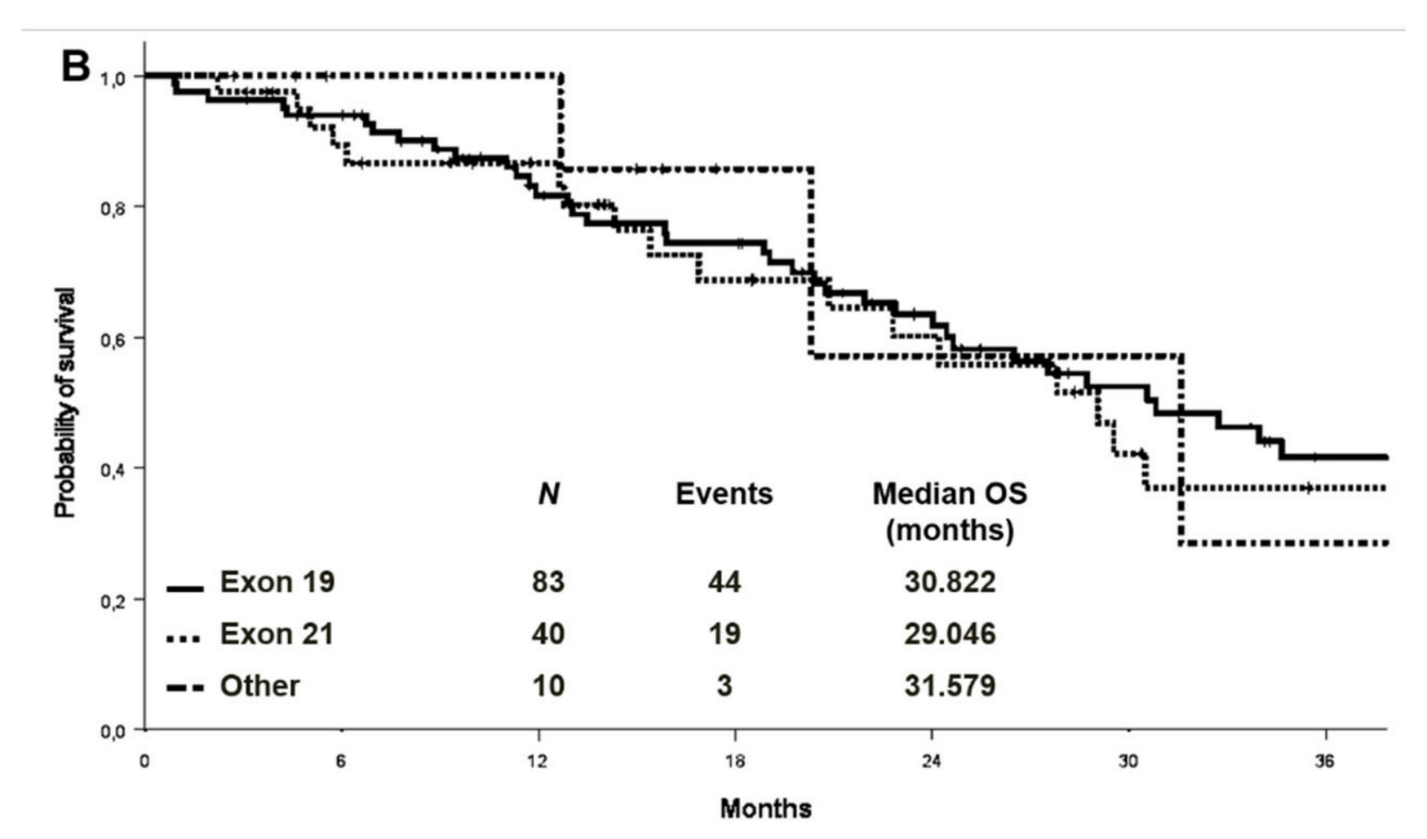
Progression-free survival (**A**) and overall survival (**B**) by EGFR mutation type in the entire cohort.

At a median follow-up of 27.9 months, the median PFS and OS were 11.2 (95% CI 9.2–13.1) and 26.5 (95% CI: 21.1–31.8) months in the cohort with complete molecular data, respectively (Figure 2). PFS and OS were also analysed by EGFR mutation type in the entire cohort (*n*:133) (Figure 3). Median PFS was 11.2, 12.1 and 11.6 months for ex19 deletion, ex21 L858R and other mutations, respectively. Median OS was 30.8, 29.0 and 31.6 months for ex19 deletion, ex21 L858R and other mutations, respectively.

The overall response rate was 68.1% in the entire cohort, 61.8% in patients treated with 1st or 2nd generation TKIs, and 70.3% in those treated with osimertinib. By analysing the type of treatment, mPFS was 10.3, 11.9 and 11.6 months in patients with exon 19 deletion, exon 21 L858R mutation, and other EGFR mutations treated with old-generation TKIs, respectively. The mPFS of patients treated with first-line osimertinib was 16.8 months and not reached in those with exon 19 deletions and L858R mutation, respectively. In the entire cohort (*n* = 133, including those without information on comutations), no significant differences were observed when comparing patients treated with first- and second-generation TKIs with those treated with upfront osimertinib (mPFS 10.6 vs. 16.8 months; HR 0.61, 95% CI 0.35–1.06; *p* = 0.081). In the subgroup of 106 patients with information on co-mutations, PFS was significantly longer with osimertinib (mPFS 16.8 vs. 9.8 months, HR 0.56, 95% CI 0.32–0.98, *p* = 0.04).

We performed univariate analysis to assess PFS and OS according to the following variables: age, ECOG PS, TKI generation, number of metastatic sites, and type of metastatic site (Table 2 and Table 3). A longer PFS was associated with third generation TKI, while shorter PFS was associated with central nervous system (CNS), bone and liver metastases. At the multivariate analysis, a significant association was maintained for TKI generation (*p* = 0.043) and presence of CNS metastases (*p* = 0.013).

Considering OS, univariate analysis showed an association between shorted OS and CNS, bone and liver metastases. At the multivariate analysis, CNS metastases were associated with shorter OS (*p* = 0.011). 

### 3.3. Association between Concomitant Alterations and Treatment Outcomes

**Figure 4:**
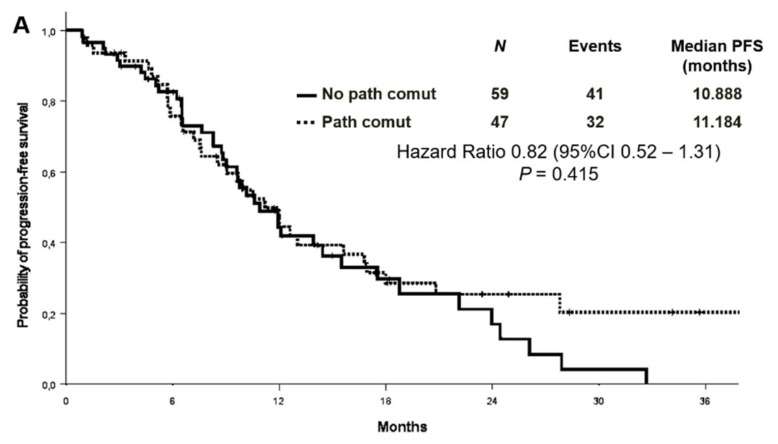

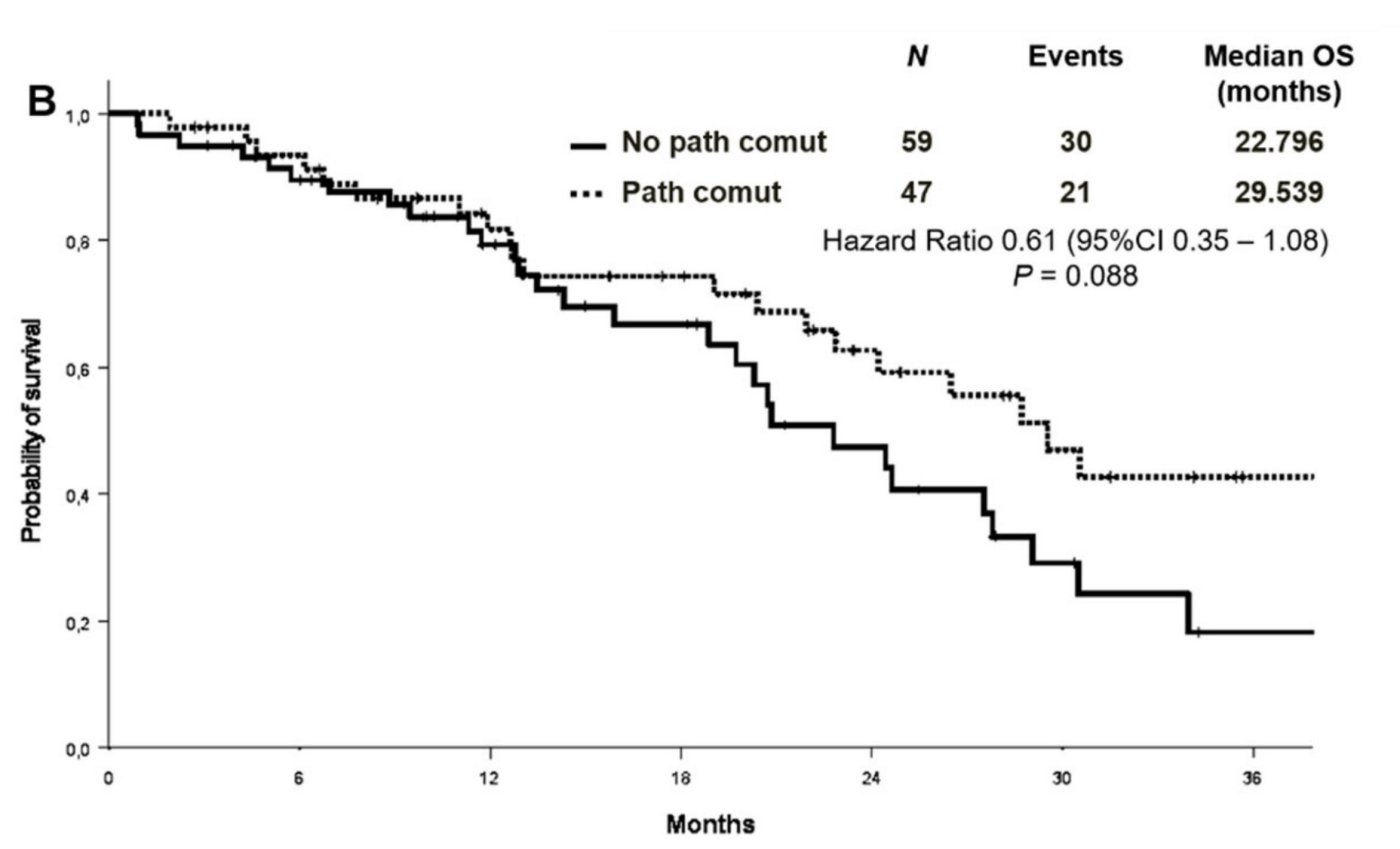
Progression-free survival (**A**) and overall survival (**B**) according to co-mutational status.

No association was found between survival and co-mutational status. The median PFS was 11.2 months in patients with concomitant alterations versus 10.9 months in patients without [Hazard Ratio (HR) 0.82 (95% CI 0.52–1.31) *p* = 0.415] (Figure 4A). OS survival was not statistically different according to the presence or absence of concomitant alterations [median OS 29.5 versus 22.8 months, respectively, HR 0.61 (95% CI 0.35–1.08) *p* = 0.088] (Figure 4B). Objective response rate (ORR) was similar between patients with or without co-occurring mutations (68.1% vs. 55.9%, *p* = 0.202).

No differences in terms of OS or PFS were found even when analysing by treatment (1st and 2nd generation TKIs or 3rd generation TKI). Indeed, median PFS was 9.9 versus 9.8 months in patients with or without co-alterations treated with old generation TKIs, respectively [HR 0.77 (95% CI 0.45–1.30) *p* = 0.319]. While mPFS was 16.8 versus 17.5 months in those treated with osimertinib [HR 1.01 (95% CI 0.37–2.73) *p* = 0.985] (Figures S2 and S3), even when analysing by treatment type.

We also performed an exploratory analysis considering all co-mutations (thus including also benign, neutral mutations and those of uncertain significance). Differences remained not statistically significant: median PFS was 10.9 months in patients without co-mutations versus 11.2 in patients with all types of mutation [HR 0.88 (95%CI 0.56–1.40) *p* = 0.597]. mOS was 20.8 versus 28.7 months, respectively [HR 0.69 (95%CI 0.40–1.21) *p* = 0.199] (Figure S4).

### 3.4. Association between Concomitant Alterations and Second-Line Treatment Outcomes 

A total of 64 patients experienced progression in the whole cohort. The most frequent sites of progression were lung (*n*:31, 48.4%), central nervous system (*n*:24, 37.5%) and pleura (*n*:15, 23.4%). Patients without co-mutations showed a significantly higher incidence of bone metastases at progression as compared to patients with co-occurring alterations (26.5% vs. 3.3%; *p* = 0.011) (Table S3). Complete molecular information, including T790M status, was obtained either by liquid or tissue biopsy in 43 patients treated with old-generation TKIs at the time of progression. T790M resistance mutation rate was similar between patients with or without baseline co-occurring mutations (52.6% vs. 70.8%; *p* = 0.220). NGS was conducted both at baseline and at the time of progression in 30 patients. No differences in molecular profile were detected in 23 (76.7%). The discordant co-mutational status included loss of *TP53*, *CTNBB1* or *PIK3CA* mutations and acquisition of *CTNBB1*, *DDR2*, *SMAD4* or *TP53* mutations. A total of 27 patients received second-line osimertinib at progression, and 10 had co-occurring mutations at baseline.

### 3.5. PD-L1 Expression and Outcomes 

**Figure 5:**
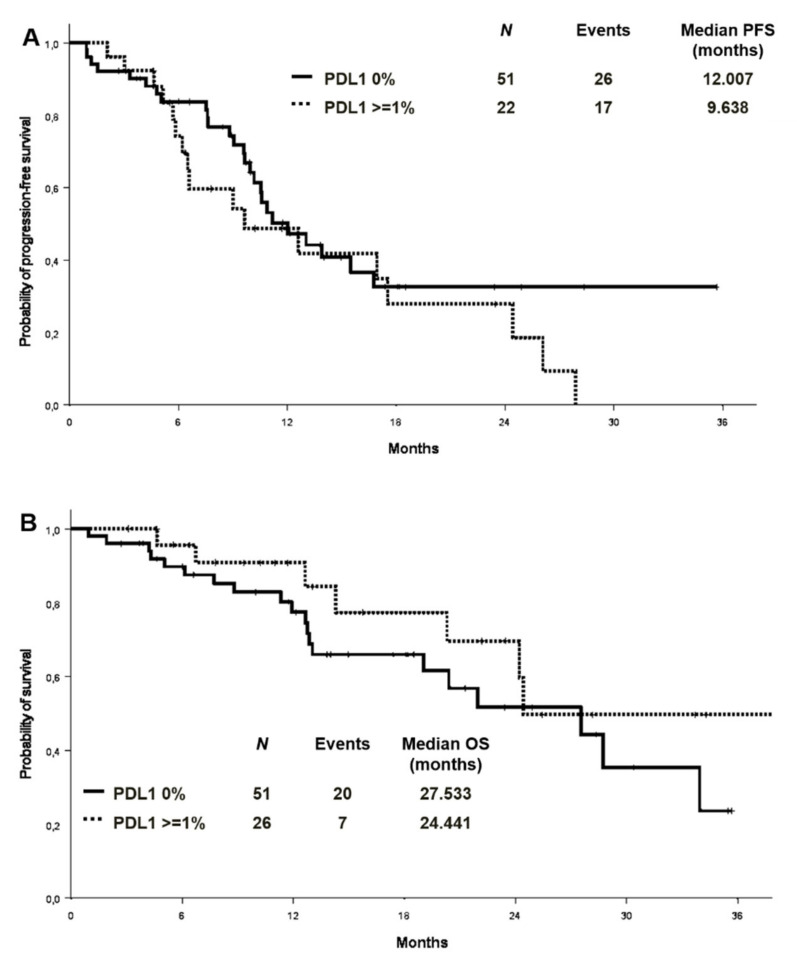
Progression-free survival (**A**) and overall survival (**B**) according to PD-L1 expression. PD-L1: programmed death-ligand 1.

PD-L1 expression level was available in 77 patients in the whole cohort, 51 of whom (66.2%) were negative. Among PD-L1 positive cases, 20 (26%) showed a TPS between 1% and 49%, and 6 (7.8%) equal or higher than 50%. An exploratory analysis on the association between PD-L1 expression and the presence/absence of co-mutations (*n*:73) showed a comparable distribution of PD-L1 expression in the two groups (Tables S4 and S5). There was no significant difference in ORR between patients with different PD-L1 expression levels (PD-L1 negative vs. PD-L1 positive: 62.7% vs. 65.4%, *p* = 0.820; PD-L1 negative vs PD-L1 1–49% vs. PD-L1 > 50%:62.7% vs. 65% vs. 66.7%; *p* = 0.972). Median PFS was 12.0 months versus 9.6 months in patients with PD-L1 of 0% and PD-L1 expression ≥1%, respectively (Figure 5A). No differences in median OS were observed in the PD-L1 negative and PD-L1 positive patients (mOS 27.5 and 24.4 months, respectively) (Figure 5B). By analysing the association between PD-L1 expression and the presence/absence of co-mutations, median PFS in PD-L1 negative patients was 11.2 months in patients with concomitant mutations versus 13.9 in patients without [HR 1.17 (95% CI 0.54–2.25) *p* = 0.694] (Figure S5). In the PD-L1 positive cohort (TPS ≥ 1%), the median PFS was 6.6 versus 17.5 months in those with and without co-mutations, respectively [HR 1.73 (95% CI 0.59–5.07) *p* = 0.318] (Figure S5).

## 4. Discussion

This retrospective study analysed data from all patients with advanced NSCLC and EGFR activating mutation treated with single-agent TKI in the first-line setting in the last 5 years at our Institutions, along with all consecutive patients treated with first-line osimertinib at Mauriziano Hospital. Patient characteristics, tumour histopathologic features, mutation types, PFS and OS are consistent with those reported in the literature. Most patients were women (65.1%) and never smokers (57.5%), and 93.4% of them had common EGFR mutations. As per other studies [3,4,5,6,7,8,9,10,11,12,13,14,15], we found that 53.8% of patients harbour concomitant alterations, even if some of them are of unknown significance or benign. Therefore, we decided to define as co-mutation positive patients with concomitant pathogenic mutations only. Interestingly, a significant correlation was found between the presence of concomitant molecular alterations and age since co-mutations occur more frequently in younger patients (<70 years old) (*p* = 0.018). This previously unreported correlation may be the epiphenomenon of the higher vulnerability of some patients to carcinogens, although we have no proof of this hypothesis. The most common co-mutated gene in our cohort was *TP53* (*n*:36, 63.2%), although only 28 were pathogenic according to the COSMIC database. Other frequent mutated genes were *CTNNB1* (6.87%), *PIK3CA* (4.9%), and others, including *NRAS*, *MET*, *PTEN*, *AKT*, *SMAD4*, *RET*, *DDR2*, *FGFR3* (9.8%). Our results suggest that genomic profile may not influence treatment efficacy and clinical outcomes of patients with advanced EGFR mutated NSCLC. The presence of concomitant alterations studied by our NGS panel was not associated with significantly different outcomes following treatment with first, second or third generation EGFR TKIs. To date, the predictive value, if any, of concomitant mutations for targeted therapy in advanced NSCLC is still a matter of study. As previously discussed, while some studies suggest that co-mutations could define a cohort with a lower probability of response to EGFR TKIs, others do not (see Table S1). Such studies are all retrospective series, with an extreme intra- and inter-study heterogeneity when dealing with ethnicity, type of *EGFR* mutations, treatment, and, more importantly, diagnostic techniques. Indeed, gene panels, as well as assay, vary between studies, and in some of them, different patients were tested with different techniques. Moreover, the definition of co-mutations or co-alterations is different among studies. The present study analysed only patients treated with single-agent TKI, carrying TKI-sensitive mutations and tested with the same technology and gene panel, thus limiting intra-study heterogeneity. However, the low number of patients included limited the statistical power of our analysis.

Different from most of the other studies reporting a higher prevalence of concomitant mutations in patients with exon 21 mutation, in our analysis, half of patients with exon 19 deletions had co-mutations as compared to 30.3% of those with exon 21 L858R mutation. Consistent with literature data [16], the most common co-mutated gene was *TP53* (63.2%) also in this cohort. Considering pathogenic mutations only, patients with co-mutations represent 44.3% of those analysed. An exploratory analysis including also benign mutations and those with unknown/neutral significance did not show any differences in ORR, PFS and OS between patients with and without other mutations. Other clinical studies have identified TP53 co-alterations as a negative prognostic marker in EGFR mutated NSCLC and a consistent predictor of worse clinical outcomes with EGFR TKI therapy [3,4,5,7,9,10,12,15]. Other results revealed that concomitant concurrence of TP53 mutation at baseline is significantly associated with shorter OS in patients treated with 1st generation TKIs but not in those treated with 2st/3nd generation ones. In addition, in the prospective randomized RELAY study, baseline *TP53* mutations were associated with shorter PFS and a trend of greater efficacy of the experimental treatment (erlotinib plus ramucirumab) was observed [17]. Moreover, patients with baseline TP53 mutations had a higher likelihood of developing T790M exon 20 mutations upon progression to both treatments. However, other studies did not show any correlation between *TP53* mutations and survival [6,8,13]. To our knowledge, very limited data exist on co-mutational profile and treatment outcomes with osimertinib, both in first or second line [14,18]. The present study shows that the benefit derived from front-line osimeritinib seems independent from the co-mutation profile. Moreover, co-mutations do not seem to predict the occurrence of T790M resistance mutations upon treatment with old-generation TKIs neither. Acquired T790M mutation was observed in 70.8% of patients without co-mutations and 52.6% of those with co-alterations (*p* = 0.220). Overall, NGS on tissue specimens was conducted both at baseline and at disease progression, without showing different molecular profiles in most cases (76.7%). This may underscore the limitations of our NGS panel to detect some relevant resistance mechanisms to TKIs. Indeed, we were not able to evaluate gene amplifications that have been already shown to have a potential prognostic and predictive role [19,20]. Interestingly, patients without concomitant alterations seem to progress more frequently to bones as compared to those with concomitant mutations (26.5% vs. 3.3%, *p* = 0.011). To further explore our cohort, we analysed PD-L1 expression levels. Several studies on the predictive role of PD-L1 expression and TKIs efficacy in EGFR-mutated NSCLC have shown conflicting results [21,22,23,24]. However, none have evaluated PD-L1 expression in relation to co-mutational profile. Our cohort showed a comparable distribution of the PD-L1 expression in the two groups. No significant differences were observed in terms of ORR, PFS and OS between patients with different PD-L1 expression levels. When dealing with PD-L1 expression and co-mutational status, neither ORR nor PFS changed according to the presence or absence of co-alterations, suggesting that PD L1 cannot be considered a predictive biomarker in this context. The main strengths of our study are the longitudinal availability of real-world clinical data, the standardised molecular profiling that was performed in the same institution using the same technology, as well as the presence of a cohort of patients treated with the first-line osimertinib. However, several limitations must be acknowledged. The retrospective design and the relatively small sample sizes of each cohort could have affected subgroup analyses. Furthermore, the small NGS panel may have missed some important molecular alterations. Moreover, tumour genetic heterogeneity is not appropriately caught by single tissue biopsy analysis [25]. Therefore, these findings require further validation within prospective studies conducted in larger cohorts.

## 5. Conclusions

This study did not demonstrate any predictive or prognostic role of co-mutation in EGFR-positive advanced NSCLC patients treated with first-line TKIs. Although, from a clinical point of view, an intra-driver diversity exists, how to identify factors explaining this phenomenon is still an open challenge. Wider diagnostic tools, both on tissue specimens and liquid biopsies, may guide treatment selection in the next-future, helping the clinician to deliver more intensive treatment strategies to high-risk patients, sparing useless toxicities in others. The development of such risk-adapted treatment algorithms requires further translational studies, especially because genomic data without clinical annotations may not bring any benefit to the patients.

## Figures and Tables

**Table 1:** Patient characteristics. ECOG: Eastern Cooperative Oncology Group; EGFR: Epidermal Growth Factor Receptor; Ex: exon; CNS: Central Nervous System.

Variable	Total (%) (*n* = 106)	Patients without Concomitant Pathogenic Mutations(%) (*n* = 59)	Patients with Concomitant Pathogenic Mutations(%) (*n* = 47)	Chi Square
Gender Male Female	37 (34.9%) 69 (65.1%)	19 (32.2%) 40 (67.8%)	18 (38.3%) 29 (61.7%)	*p* = 0.513
**Age (median, range)**	69.8 (32.0–90.7)	72.5 (34.6–90.7)	66.6 (32.0–85.5)	
**Age** Younger than 70 years 70 years or older	54 (50.9%) 52 (49.1%)	24 (40.7%) 35 (59.3%)	30 (63.8%) 17 (36.2%)	***p* = 0.018**
**ECOG Performance Status** PS 0 PS 1 PS 2 PS 3	48 (45.3%) 50 (47.2%) 7 (6.6%) 1 (0.9%)	25 (42.4%) 27 (45.8%) 6 (10.2%) 1 (1.7%)	23 (48.9%) 23 (48.9%) 1 (2.1%) 0	*p* = 0.300
**Smoking status** Never Former/current	61 (57.5%) 45 (42.5%)	35 (59.3%) 24 (40.7%)	26 (55.3%) 21 (44.7%)	*p* = 0.679
**Stage at diagnosis** Stage I Stage II Stage III Stage IV	1 (0.9%) 6 (5.7%) 5 (4.9%) 94 (88.7%)	0 3 (5.1%) 2 (3.4%) 54 (91.5%)	1 (2.1%) 3 (6.4%) 3 (6.4%) 40 (85.1%)	*p* = 0.583
**Histology** Adenocarcinoma Non-adenocarcinoma	105 (99.1%) 1 (0.9%)	58 (98.3%) 1 (1.7%)	47 (100.0%) 0	*p* = 0.370
**EGFR mutation** Ex19 del Ex21 L858R Rare or double	66 (62.3%) 33 (31.1%) 7 (6.6%)	33 (55.9%) 23 (39.0%) 3 (5.1%)	33 (70.2%) 10 (21.3%) 4 (8.5%)	*p* = 0.138
**Treatment** Gefitinib/Erlotinib/Afatinib Osimertinib	65 (61.3%) 41 (38.7%)	34 (57.6%) 25 (42.4%)	31 (66.0%) 16 (34.0%)	*p* = 0.382
**Number of metastatic sites** 0 sites 1 site 2 sites 3 sites More than 3 sites	1 (0.9%) 35 (33.0%) 29 (27.4%) 22 (20.7%) 19 (18.0%)	0 20 (33.9%) 13 (22.0%) 14 (23.7%) 12 (20.3%)	1 (2.1%) 15 (31.9%) 16 (34.0%) 8 (17.0%) 7 (14.9%)	*p* = 0.637
**Metastatic sites at diagnosis** Lung Pleural CNS Liver Bone Adrenal Nodes	32 (30.2%) 47 (44.3%) 35 (33.0%) 18 (17.0%) 51 (48.1%) 14 (13.2%) 53 (50%)	19 (32.2%) 33 (55.9%) 20 (33.9%) 12 (20.3%) 29 (49.2%) 7 (11.9%) 24 (40.7%)	13 (27.7%) 14 (29.8%) 15 (31.9%) 6 (12.8%) 22 (46.8%) 7 (14.9%) 29 (61.7%)	*p* = 0.613 ***p* = 0.007** *p* = 0.829 ***p* = 0.302** *p* = 0.810 *p* = 0.647 *p* = 0.032

**Table 2:** Univariate and multivariate analysis for progression-free survival. Bold indicates statistically significant findings (*p* < 0.05).

Progression-Free Survival		
Variable	Univariate Analysis Hazard Ratio (95%CI), *p*-Value	Multivariate Analysis Hazard Ratio (95%CI), *p*-Value
**Pathogenic comutations (yes vs. no)**	0.82 (0.52–1.31), *p* = 0.415	
**Age (older than 70 vs. younger)**	0.84 (0.53–1.33), *p* = 0.451	
**ECOG performance status (>=1 vs. 0)**	1.36 (0.85–2.18), *p* = 0.196	
**TKI generation (osimertinib vs. other)**	**0.56 (0.32–0.98),** ***p*** **=** **0.04**	**0.55 (0.31–0.98), *p* = 0.043**
**Number of metastatic sites (>1 vs. 1)**	1.50 (0.92–2.46), *p* = 0.106	
**Lung (yes vs. no)**	0.91 (0.53–1.57), *p* = 0.735	
**Pleura (yes vs. no)**	0.69 (0.43–1.10), *p* = 0.116	
**CNS (yes vs. no)**	**2.43 (1.49–3.96), *p* < 0.001**	**2.14 (1.17–3.89), *p* = 0.013**
**Liver (yes vs. no)**	**1.92 (1.02–3.61),** ***p*** **=** **0.044**	
**Bone (yes vs. no)**	**1.62 (1.02–2.57),** ***p*** **=** **0.042**	

**Table 3:** Univariate and multivariate analysis for overall survival. Bold indicates statistically significant findings (*p* < 0.05)

Overall Survival		
Variables	Univariate Analysis Hazard Ratio (95%CI), *p*-Value	Multivariate Analysis Hazard Ratio (95%CI), *p*-Value
**Pathogenic comutations (yes vs. no)**	0.61 (0.35–1.08), *p* = 0.088	
**Age (older than 70 vs. younger)**	1.25 (0.71–2.18), *p* = 0.439	
**ECOG performance status (>=1 vs. 0)**	1.72 (0.97–3.04), *p* = 0.062	
**TKI generation (osimertinib vs. other)**	0.86 (0.40–1.86), *p* = 0.698	
**Number of metastatic sites (>1 vs. 1)**	1.58 (0.85–2.95), *p* = 0.150	
**Lung (yes vs. no)**	1.01 (0.52–1.93), *p* = 0.989	
**Pleura (yes vs. no)**	0.79 (0.45–1.40), *p* = 0.421	
**CNS (yes vs. no)**	**2.08 (1.20–3.62),** ***p*** **=** **0.009**	**2.62 (1.25–5.46),** ***p*** **=** **0.011**
**Liver (yes vs. no)**	**2.17 (1.10–4.29),** ***p*** **=** **0.025**	
**Bone (yes vs. no)**	**1.98 (1.12–3.48),** ***p*** **=** **0.018**	

## Data Availability

The data presented in this study are available on request from the corresponding authors. The data are not publicly available due to privacy and ethical reasons.

## Supplementary Materials

The following are available online at https://www.mdpi.com/article/10.3390/cancers13102425/s1, Figure S1: Study consort diagram, Figure S2: Progression-free survival according to the type of EGFR TKI, Figure S3: Overall survival according to the type of EGFR TKI, Figure S4: Survival according to co-mutational status, considering all co-mutations, Figure S5: Progression-free survival in PD-L1 positive and negative patients by co-mutational status. PD-L1: Programmed death-ligand 1, Table S1: Studies evaluating in advanced EGFR+ NSCLC, Table S2: Co-mutational status by EGFR mutation type, Table S3: Site of progression according to co-mutational status, Table S4: Distribution of PD-L1 expression in patients with and without concomitant mutations. PD-L1: Programmed death-ligand 1, Table S5: Distribution of PD-L1 expression levels in patients with and without co-mutations. PD-L1: Programmed death-ligand 1.

## References

[1] Word Health Organization International Agency for Research on Cancer. https://gco.iarc.fr/today/online-analysis-table?v=2020&mode=cancer&mode_population=continents&population=900&populations=900&key=asr&sex=0&cancer=39&type=1&statistic=5&prevalence=0&population_group=0&ages_group%5B%5D=0&ages_group%5B%5D=17&group_cancer=1&include_nmsc=1&include_nmsc_other=1.

[2] Greenhalgh J., Boland A., Bates V., Vecchio F., Dundar Y., Chaplin M., Green J.A. (2021). First-line treatment of advanced epidermal growth factor receptor (EGFR) mutation positive non-small cell lung cancer. Cochrane Database Syst Rev..

[3] Barnet M.B., O’Toole S., Horvath L.G., Selinger C., Yu B., Ng C.C., Boyer M., Cooper W.A., Kao S. (2017). EGFR-co-mutated advanced NSCLC and response to EGFR tyrosine kinase inhibi-tors. J. Thorac. Oncol..

[4] Hu W., Liu Y., Chen J. (2017). Concurrent gene alterations with EGFR mutation and treatment efficacy of EGFR-TKIs in Chinese pa-tients with non-small cell lung cancer. Oncotarget.

[5] Labbé C., Cabanero M., Korpanty G.J., Tomasini P., Doherty M.K., Mascaux C., Jao K., Pitcher B., Wang R., Pintilie M. (2017). Prognostic and predictive effects of TP53 co-mutation in patients with EGFR -mutated non-small cell lung cancer (NSCLC). Lung Cancer.

[6] VanderLaan P.A., Rangachari D., Mockus S.M., Spotlow V., Reddi H.V., Malcolm J., Huberman M.S., Joseph L.J., Kobayashi S.S., Costa D.B. (2017). Mutations in TP53, PIK3CA, PTEN and other genes in EGFR mutated lung cancers: Correlation with clinical outcomes. Lung Cancer.

[7] Hong S., Gao F., Fu S., Wang Y., Fang W., Huang Y., Zhang L. (2018). Concomitant genetic alterations with response to treatment and epidermal growth factor recep-tor tyrosine kinase inhibitors in patients with EGFR-mutant advanced non-small cell lung cancer. JAMA Oncol..

[8] Jakobsen J.N., Santoni-Rugiu E., Grauslund M., Melchior L., Sørensen J.B. (2018). Concomitant driver mutations in advanced EGFR-mutated non-small-cell lung cancer and their impact on erlotinib treatment. Oncotarget.

[9] Helena A.Y., Suzawa K., Jordan E.J., Zehir A., Ni A., Kim H.R., Kris M.G., Hellmann M.D., Li B.T., Somwar R. (2018). Concurrent Alterations in EGFR-Mutant Lung Cancers Associated with Resistance to EGFR Kinase Inhibitors and Characterization of MTOR as a Mediator of Resistance. Clin. Cancer Res..

[10] Kim Y., Lee B., Shim J.H., Lee S.H., Park W.Y., Choi Y.L., Sun J.M., Ahn J.S., Ahn M.J., Park K. (2019). Concurrent genetic alterations predict the progression to target therapy in EGFR-mutated ad-vanced NSCLC. J Thorac. Oncol..

[11] Chang S.-C., Lai Y.-C., Chang C.-Y., Huang L.-K., Chen S.-J., Tan K.T., Yu P.-N., Lai J.-I. (2019). Concomitant Genetic Alterations are Associated with Worse Clinical Outcome in EGFR Mutant NSCLC Patients Treated with Tyrosine Kinase Inhibitors. Transl. Oncol..

[12] Chen M., Xu Y., Zhao J., Zhong W., Zhang L., Bi Y., Wang M. (2019). Concurrent Driver Gene Mutations as Negative Predictive Factors in Epidermal Growth Factor Receptor-Positive Non-Small Cell Lung Cancer. EBioMedicine.

[13] Rachiglio A.M., Fenizia F., Piccirillo M.C., Galetta D., Crinò L., Vincenzi B., Barletta E., Pinto C., Ferraù F., Lambiase M. (2019). The Presence of Concomitant Mutations Affects the Activity of EGFR Tyrosine Kinase Inhibitors in EGFR-Mutant Non-Small Cell Lung Cancer (NSCLC) Patients. Cancers.

[14] Cheng Y., Ma L., Liu Y., Zhu J., Xin Y., Liu X., Wang Y., Zhang T., Yang C., Wang S. (2020). Comprehensive characterization and clinical impact of concomitant genomic alterations in EGFR-mutant NSCLCs treated with EGFR kinase inhibitors. Lung Cancer.

[15] Christopoulos P., Kirchner M., Roeper J., Saalfeld F., Janning M., Bozorgmehr F., Magios N., Kazdal D., Volckmar A.L., Brückner L.M. (2020). Risk stratification of EGFR+ lung cancer diagnosed with pane-based next-generation sequencing. Lung Cancer.

[16] Skoulidis F., Heymach J.V. (2019). Co-occurring genomic alterations in non-small-cell lung cancer biology and therapy. Nat. Rev. Cancer.

[17] Garon E., Reck M., Nishio K., Heymach J.V., Nishio M., Novello S., Paz-Ares L., Popat S., Aix S.P., Wijayawardana S. (2020). Abstract CT215: RELAY, ramucirumab plus erlotinib (RAM+ERL) versus placebo plus erlotinib (PBO+ERL) in previously untreatedEGFRmutation-positive metastatic NSCLC: Next generation sequencing (NGS) results. Tumor Biology.

[18] Jiang W., Zeng A., Ning R., Zhao W., Su C., Wang H., Zhou S., Yu Q. (2020). Predictive value of tumor genetic alteration profiling for chemotherapy and EGFR-TKI treatment in advanced NSCLC. Oncol. Lett..

[19] Zhang X., Zhang Y., Tang H., He J. (2016). EGFR gene copy number as a predictive/biomarker for patients with non-small-cell lung cancer receiving tyrosine kinase inhibitor treatment: A systematic review and meta-analysis. J. Investig. Med..

[20] Dahabreh I.J., Linardou H., Kosmidis P., Bafaloukos D., Murray S. (2011). EGFR gene copy number as a predictive biomarker for pa-tients receiving tyrosine kinase inhibitor treatment: A systematic review and meta-analysis in non-small-cell lung cancer. Ann. Oncol..

[21] Takada K., Toyokawa G., Tagawa T., Kohashi K., Shimokawa M., Akamine T., Takamori S., Hirai F., Shoji F., Okamoto T. (2018). PD-L1 expression according to the EGFR status in primary lung adenocarcinoma. Lung Cancer.

[22] Hsu K.-H., Huang Y.-H., Tseng J.-S., Chen K.-C., Ku W.-H., Su K.-Y., Chen J.J., Chen H.-W., Yu S.-L., Yang T.-Y. (2019). High PD-L1 expression correlates with primary resistance to EGFR-TKIs in treatment naïve advanced EGFR-mutant lung adenocarcinoma patients. Lung Cancer.

[23] Yang C.Y., Liao W.Y., Ho C.C., Chen K.Y., Tsai T.H., Hsu C.L., Su K.Y., Chang Y.L., Wu C.-T., Hsu C.-C. (2020). Association between programmed death-ligand 1 expression, immune microenviron-ments, and clinical outcomes in epidermal growth factor receptor mutant lung adenocarcinoma patients treated with tyro-sine kinase inhibitors. Eur. J. Cancer.

[24] Liu J., Itchins M., Nagrial A., Cooper W.A., De Silva M., Barnet M., Varikatt W., Sivasubramaniam V., Davis A., Gill A.J. (2021). Relationship between PD-L1 expression and outcome in EGFR-mutant lung cancer patients treated with EGFR tyrosine kinase inhibitors. Lung Cancer.

[25] Almendro V., Marusyk A., Polyak K. (2013). Cellular Heterogeneity and Molecular Evolution in Cancer. Annu. Rev. Pathol. Mech. Dis..

