# Supplementary material for: Clinical and Molecular Features of Epidermal Growth Factor Receptor (EGFR) Mutation Positive Non-Small-Cell Lung Cancer (NSCLC) Patients Treated with Tyrosine Kinase Inhibitors (TKIs): Predictive and Prognostic Role of Co-Mutations
Source: Cancers (Basel). 2021 May 17;13(10):2425. doi: 10.3390/cancers13102425 (PMC8156829; doi:10.3390/cancers13102425)
Supplement: Supplementary file 1 [file cancers-13-02425-s001.zip › cancers-1209888-supplementary.pdf]

cancers

MDPI

*Supplementary Materials*

# Clinical and Molecular Features of Epidermal Growth Factor Receptor (EGFR) Mutation Positive Non-Small-Cell Lung Cancer (NSCLC) Patients Treated with Tyrosine Kinase Inhibitors (TKIs): Predictive and Prognostic Role of Co-Mutations

**Paolo Bironzo [1,*,†], Maria Lucia Reale [1,*,†], Tessa Sperone [1], Fabrizio Tabbò [1], Andrea Caglio [2], Angela Listì [3], Francesco Passiglia [1], Massimo Di Maio [2], Luisella Righi [3], Federico Bussolino [4,5], Giorgio V. Scagliotti [1] and Silvia Novello [1]**

1 Thoracic Oncology Unit, Department of Oncology, San Luigi Gonzaga Hospital, University of Torino, Orbassano 10043, Italy; tessa.sperone@edu.unito.it (T.S.), fabrizio.tabbo@unito.it (F.T.), francesco.passiglia@unito.it (F.P.), giorgio.scagliotti@unito.it (G.V.S.), silvia.novello@unito.it (S.N.)
2 Medical Oncology Unit, Department of Oncology, Mauriziano Umberto I Hospital, University of Torino, Torino 10128, Italy; andrea.caglio@edu.unito.it (A.C.), massimo.dimaio@unito.it (M.D.M.)
3 Pathology Unit, Department of Oncology, San Luigi Gonzaga Hospital, University of Torino, Orbassano 10043, Italy; alisti@live.it (A.L.), luisella.righi@unito.it (L.R.)
4 Department of Oncology, University of Torino, Candiolo, 10060, Italy; federico.bussolino@unito.it
5 Laboratory of Vascular Oncology, Candiolo Cancer Institute-IRCCS-FPO, Candiolo, 10060, Italy
* Correspondence: paolo.bironzo@unito.it (P.B.); realemarialucia@gmail.com (M.L.R.)
† Authors equally contributed to the work.

## Supplementary materials

*List of rare and double mutations (8/106):*

Exon 18 G719A (n:3), Exon 18 G719S (n:1), Exon 19 L747P (n:1), Exon 21 L861Q (n:1), Exon 18 E709K + Exon 21 L858R (n:1), Exon 18 G719S + Exon 20 S768I (n:1)

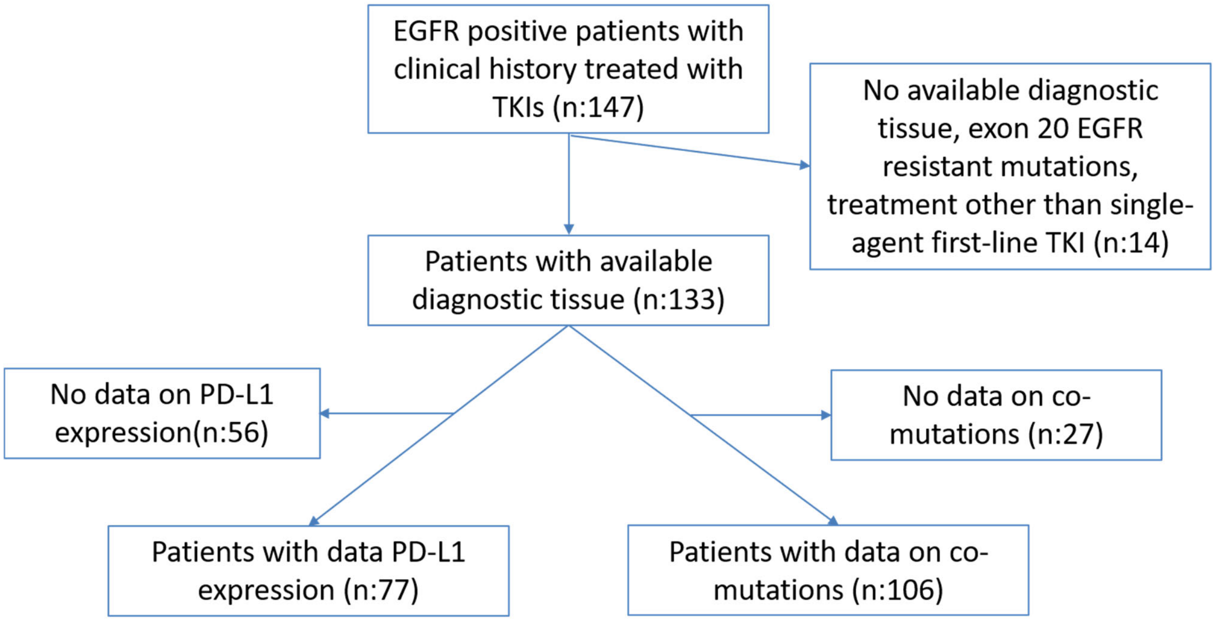


**Figure S1.** Consort diagram. EGFR: Epidermal Growth Factor Receptor; TKI: Tyrosine Kinase Inhibitor; PD-L1: Programmed Death-Ligand 1.

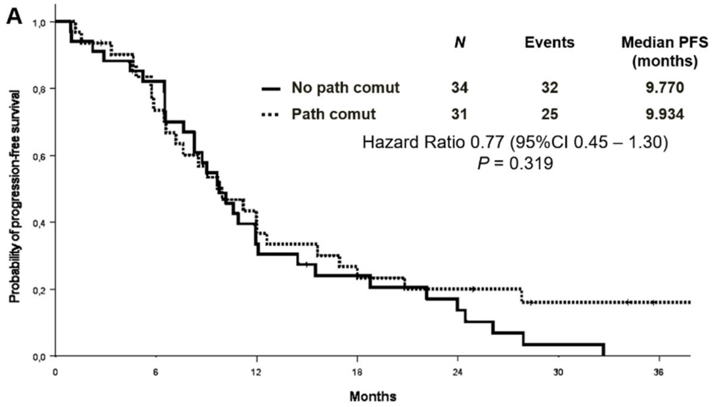


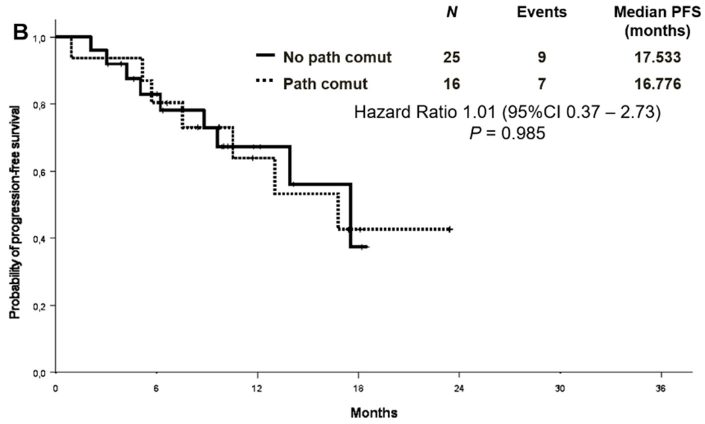

**Figure S2.** Progression-free survival according to the type of EGFR TKI in patients with and without concomitant mutaTable 3. Overall survival according to the type of EGFR TKI in patients with and without concomitant mutations: first/second generation (A) and third generation (B). EGFR: Epidermal Growth Factor Receptor. TKI: Tyrosine Kinase Inhibitor.

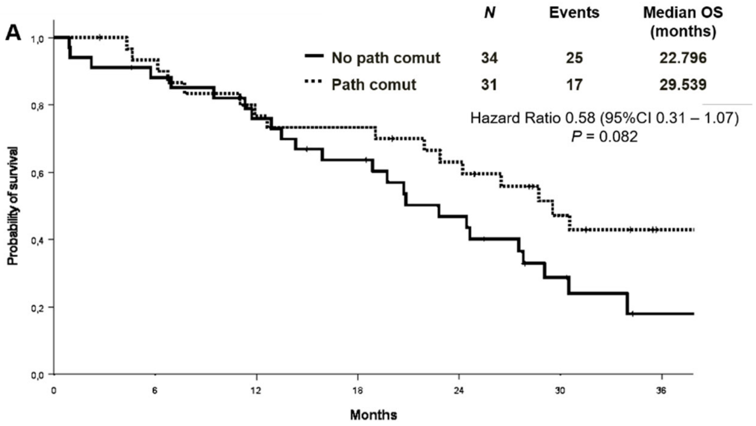


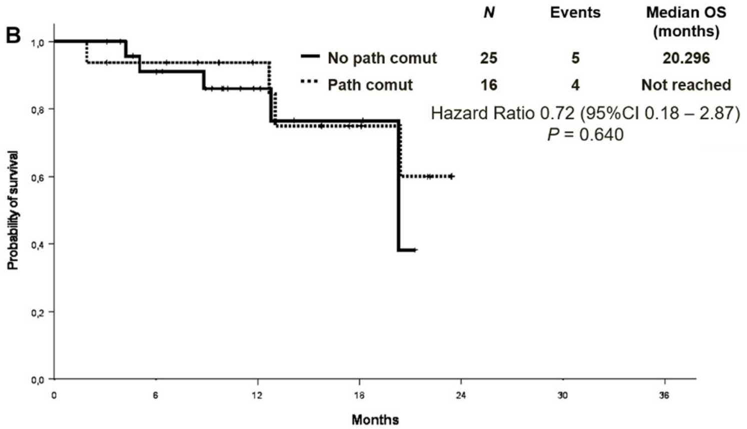


**Figure S3.** Overall survival according to the type of EGFR TKI in patients with and without concomitant mutations: first/second generation (A) and third generation (B). EGFR: Epidermal Growth Factor Receptor. TKI: Tyrosine Kinase Inhibitor.

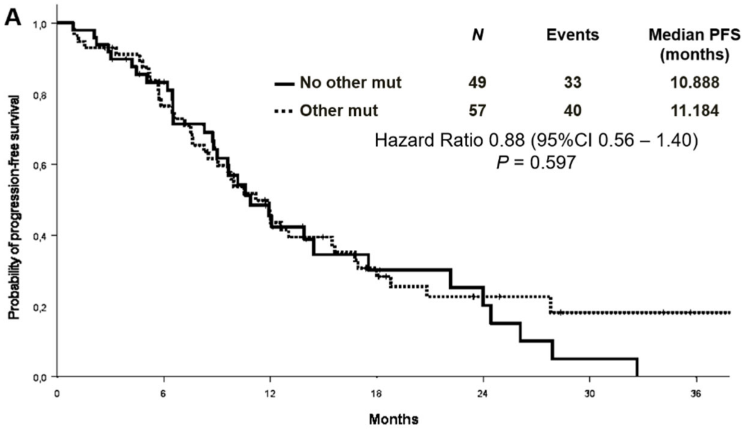

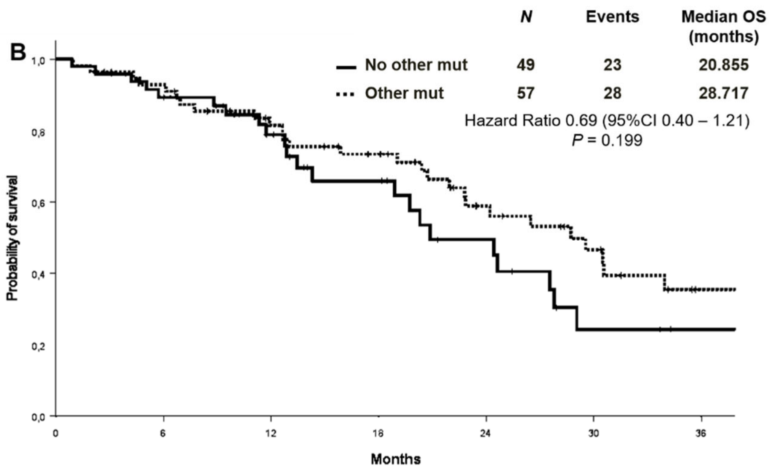


**Figure S4.** Progression-free survival (A) and overall survival (B) according to co-mutational status, considering all co-mutations (pathogenic and non-pathogenic/ of unknown significance).

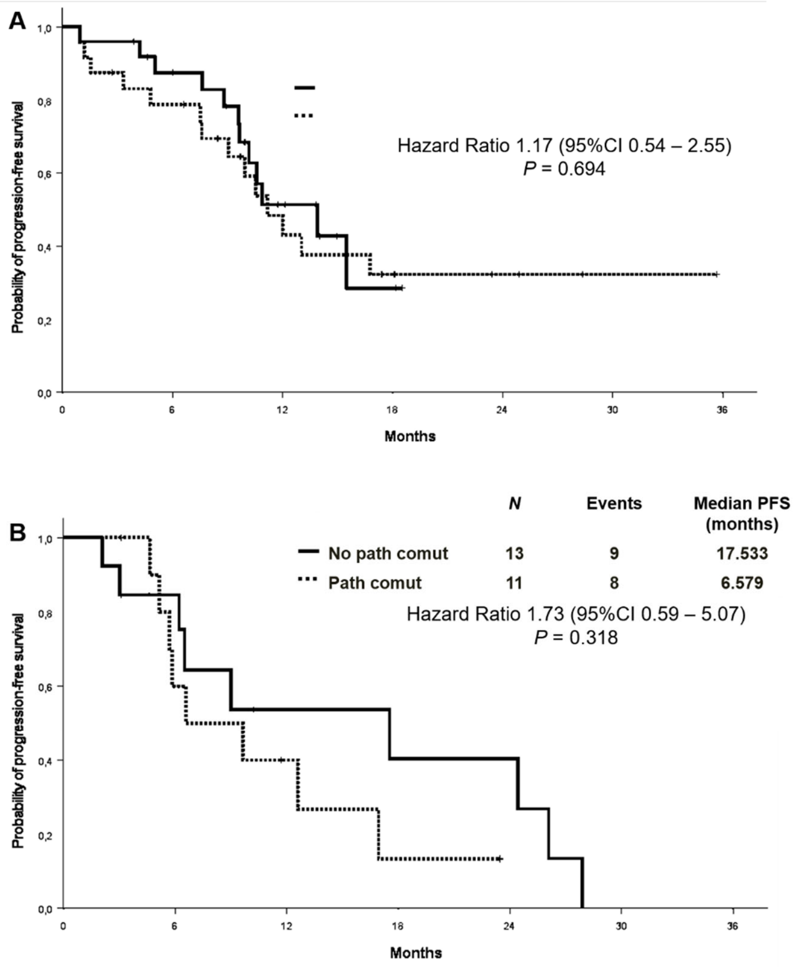


**Figure S5.** Progression-free survival in PD-L1 positive and negative patients by co-mutational status. PD-L1: Programmed death-ligand 1.

**Table S1.** Studies evaluating co-mutations in advanced NSCLC patients with activating EGFR mutations. EGFR: epidermal growth factor receptor; NSCLC: non-small cell lung cancer; TKI: tyrosine kinase inhibitors; NGS: next-generation sequencing; HR: hazard ratio; CI: confidence interval; CNV: copy number variation; PFS: progression-free survival; OS: overall survival; ORR: objective response rate; IHC: immunohistochemistry; FISH: fluorescence *in situ* hybridization.

| Author, year | Type of study, Country | Number of advanced patients analysed | Type of first-line treatment (number treated) | Type of test | Most frequent concurrent alterations | Outcomes |
|---|---|---|---|---|---|---|
| Barnet, 2017[3] | Single-centre, retrospective, Australia | 62 | 1st and 2nd generation TKIs (n: 62) | 19 oncogenes, MassArray (OncoCarta v1.0 panel) | *EGFR* (8%) and *PIK3CA* (3.2%) mutations | Significantly shorter mPFS in patients with co-mutations (5.7 vs 12.3 months; p=0.02) and lower ORR (38% vs 89%, p<0.001) |
| Hu, 2017[4] | Single-centre, retrospective, China | 320 (stage IIIB and IV) | 1st or 2nd generation TKIs (n: 320) | PCR for *HER2*, *KRAS*, *NRAS*, *BRAF*, *PIK3CA* mutations and for *ALK*, *ROS1* and *RET* rearrangements | *PIK3CA* (2.8%) and *KRAS* (0.9%) mutations, *ALK* (1.9%), *RET* (0.8%) and *ROS1* (0.8%) rearrangements | Concomitant mutations are associated with significantly shorter PFS; no difference in OS were observed |
| Labbé, 2017[5] | Single-centre, retrospective, Canada, focused on *TP53* only | 60 | 1st generation TKIs (n: 60) | *TP53* Sanger sequencing or NGS | *TP53* 56% (missense: 17%) | Significantly shorter mPFS in patients with *TP53* missense mutations (HR 1.91, 95% CI 1.01-3.60, p=0.04) |
| VanderLaan, 2017[6] | Single-centre, retrospective, United States | 20 | 1st or 2nd generation EGFR TKIs (n: 16) | SNaPshot-NGS or JAX-Cancer Treatment Profile | *TP53* (50%), *PIK3CA* (10%), *PTEN* (5%) mutations | No significant differences between patients with and without co-mutations |
| Hong, 2018[7] | Single-centre, retrospective, China | 58 | 1st generation TKIs (n: 58) | 49 cancer-related genes with Ion Pi Sequencing 200 kit v2 (Thermo Fisher Scientific) | *TP53* (41.4%), *EGFR* T790M (13.8%), *KRAS* (6.9%), *PIK3CA* (5.2%) mutations | Concomitant mutations were associated with lower ORR, and shorter PFS and OS |

| | | | | | | |
|---|---|---|---|---|---|---|
| Jakobsen, 2018[8] | Single-centre, retrospective, Denmark | 18 (+ 5 with stage IIIA/B) | 1st generation TKIs (n: 23) | 22 lung/colon-cancer associated genes (Ion AmpliSeq Colon—Lung Cancer Research Panel v2 on Ion Torrent, Thermo Fisher Scientific); *MET* IHC and FISH, and *ALK* IHC were also performed | *TP53* (67%), *CTNNB1* (13%). 5 and 4 samples had *MET* overexpression and amplification, respectively. | No differences in terms of PFS or OS between patients with or without co-alterations |
| Yu, 2018[9] | Single-centre, retrospective, United States | 200 | 1st or 2nd generation EGFR TKIs (n: 200) | NGS: MSK Impact, version 1, 2 or 3 | *TP53* (60%), *PIK3CA* (12%), *CTNNB1* (9%), *RB1* (10%) mutations, and *EGFR* (22%), *TTF1*(15%), *MDM2 (12%), CDK4* (10%) and *FOXA1* (10%) amplifications | Shorter time to progression on TKI associated with *ERBB2* (HR 2.42, p=0.018) and *MET* (HR 3.65, p=0.029) amplifications, and *TP53* mutations (HR 1.68, p=0.006) |
| Kim, 2019[10] | Single-centre, retrospective, Korea | 75 | 1st or 2nd generation EGFR TKIs (n: 75) | NGS: CancerSCAN panel, version 1 or 2 | *TP53* (57.3%), *CTNNB1* (9.3%), *PIK3CA* (8%), *RB1*(6.7%) mutations | *TP53* mutations independently associated with worse PFS (HR 2.02, 95% CI 1.04-3.93; p=0.038) |
| Chang, 2019[11] | Single-centre, retrospective, Taiwan | 33 | 1st and 2nd generation TKIs (n: 33) | ACTonco®+ panel using Ion Proton sequencer with Ion PI chip (Life Technologies) | *TP53* (32%) mutations, *CDK4* (26%) and *CDKN2A* (23%) alterations (mainly CNV gain or loss) | FGFR3 mutations and CDKN2A CNV loss associated with shorter PFS; patients with any concomitant mutations have worse OS (24.1 vs 40.8 momths; p=0.029) |
| Chen, 2019[12] | Singe-centre, retrospective, China | 71 (selected according to PFS ≤6 months or ≥24 months) | 1st generation TKIs (n: 71) | 416 cancer-relevant genes (Illumina Hiseq 4000 NGS platforms) | *TP53* (51%), *MAP2K2* (15%), *NKX2-1* (15%), *CTNNB1* (15%), *RB1* (12%) mutations, *EGFR* amplification (18%). | *TP53* missense and *PIK3CA* missense mutations more frequent in the short PFS group as well as co-occurring driver mutations (*ALK* rearrangement, *MET* amplification, *BRAF V600E* mutation). No difference in *TP53* mutation rate between short and long PFS group. |

| | | | | | | |
|---|---|---|---|---|---|---|
| Rachiglio, 2019[13] | Multicentre, retrospective, Italy | 133 | 1st and 2nd generation TKIs (n: 133) | 22 cancer-related genes panel (Ion AmpliSeq Colon—Lung Cancer Panel on Ion Torrent, Thermo Fisher Scientific) | *TP53* (17.3%), *KRAS* (14%), *PIK3CA* (9%), *EGFR* T790M (6.8%) mutations | Concomitant mutations (but not *TP53*) are associated with significantly shorter PFS on EGFR TKIS |
| Cheng, 2020[14] | Single-centre, retrospective, China | 175 (stage IIIA or higher) 160 with EGFR mutations | 1st, 2nd, 3rd generation TKIs +/- chemotherapy or anti-angiogenics (n: 110, 1st; n: 35, 2nd; n: 15, 3rd ) | 520 or 168 cancer-related gene panel based on NextSeq 500 (Illumina technology) | *TP53* (57%), *PIK3CA* (6%), *PMS2* (6%), *DMT3A* (6%), *APC* (6%), *MYC* (6%) mutations. 6.9% other driver alterations (*ERBB2* and *MET* amplifications, *ERBB2*, *BRAF* and *KRAS* mutations) | *TP53* mutation, *ERBB2* and *FGF19* amplifications associated with significantly worse OS upon treatment with 1st but not 2nd generation TKIs; CNV associated with significantly shorter PFS on 3rd generation TKIs |
| Christopoulos, 2020[15] | Multi-centre, retrospective, Germany | 261 | 1st and 2nd generation TKIs (n: 219) | 38-42 genes custom panel (NGS, ThermoFisher Scientific) | *TP53* (44%), *CTNNB1* (4.6%) | *TP53* mutations independently associated with PFS and OS |

**Table S2.** Co-mutational status by EGFR mutation type. EGFR: Epidermal Growth Factor Receptor.

| | Patients without concomitant pathogenic mutations (n=59) | Patients with concomitant pathogenic mutations (n=47) | All patients (n=106) |
|---|---|---|---|
| **Main mutation** | | | |
| Exon 19 | 33 (55.9%) | 33 (70.2%) | 66 |
| L858R | 23 (39.0%) | 10 (21.3%) | 33 |
| Other | 3 (5.1%) | 4 (8.5%) | 7 |
| Chi square p=0.138 | | | |

**Table S3.** Site of progression according to co-mutational status.

| | Patients without concomitant pathologic mutations (n=34) | Patients with concomitant pathologic mutations (n=30) | Chi square |
|---|---|---|---|
| Lung | 18/34 (52.9%) | 13/30 (43.3%) | p=0.443 |
| Pleural | 10/34 (29.4%) | 5/30 (16.7%) | p=0.230 |
| CNS | 12/34 (35.3%) | 12/30 (40.0%) | p=0.698 |
| Liver | 7/34 (20.6%) | 4/30 (13.3%) | p=0.443 |
| Bone | 9/34 (26.5%) | 1/30 (3.3%) | **p=0.011** |
| Adrenal | 1/34 (2.9%) | 2/30 (6.7%) | p=0.482 |
| Nodes | 4/34 (11.8%) | 1/30 (3.3%) | p=0.210 |

**Table S4.** Distribution of PD-L1 expression in patients with and without concomitant mutations. PD-L1: Programmed death-ligand 1.

| | Patients without concomitant pathologic mutations (n=38) | Patients with concomitant pathologic mutations (n=35) | Wilcoxon – Mann-Whitney |
|---|---|---|---|
| Median | 0 | 0 | p=0.916 |
| Range | 0 - 75 | 0 - 95 | |

**Table S5.** Distribution of PD-L1 expression levels in patients with and without co-mutations. PD-L1: Programmed death-ligand 1.

| | Patients without concomitant pathologic mutations (n=38) | Patients with concomitant pathologic mutations (n=35) | Chi square |
|---|---|---|---|
| PD-L1 0% | 25 (65.8%) | 24 (68.6%) | p=0.483 |
| PD-L1 1-49% | 11 (28.9%) | 7 (20.0%) | |
| PD-L1 >=50% | 2 (5.3%) | 4 (11.4%) | |
